# *SNORA71C* promotes development and metastasis of breast cancer by regulating *RUNX1* and ferroptosis

**DOI:** 10.1002/mco2.262

**Published:** 2023-04-17

**Authors:** Bumin Xie, Xi Chen, Lin Zhao

**Affiliations:** ^1^ Department of Breast Surgery Cancer Hospital of Dalian University of Technology Liaoning Cancer Hospital & Institute Shenyang Liaoning Province People's Republic of China

Dear Editor,

Breast cancer (BC) is the most common cancer in women and the second leading cause of cancer‐related death worldwide after lung cancer.^1^ At the molecular level, it is divided into subtypes based on the binding (presence or absence) of estrogen receptor (ER), progesterone receptor (PR), and human epidermal growth factor receptor 2 (HER2). The prognosis and treatment strategy of BC are determined by the expression of these receptors. Despite the advances in early diagnosis and treatment effectiveness, this study aims to discover tumor markers and new therapeutic strategies.

snoRNAs are a group of small noncoding RNAs that plays an important role in the occurrence, development, and metabolism of a variety of tumor cells.^2^ However, the involvement of snoRNA in BC development has not been completely elucidated.

We have analyzed the differential expression of snoRNA in BC tissues in the Cancer Genome Atlas and established that the expression of small nucleolar RNA *SNORA71C* (*SNORA71C*) in BC tissues was two‐fold higher than that of normal breast tissues. The differential expression of snoRNA was identified by combining fold change (Figure 1A). *SNORA71C*‐ containing specimens from 47 BC patients were analyzed by Real‐time reverse transcription PCR (qRT‐PCR); the normal tissues around the tumor tissues were used as the control. The results suggested that the expression level of *SNORA71C* in BC tissues was significantly higher than that in normal breast tissue (Figure 1B). In tumor tissues, the expression of *SNORA71C* was not related to age, tumor diameter, lymph node metastasis, Ki67, ER, PR, HER2, and cancer grade (Table S1). We also found that *SNORA71C* expression in normal breast cells MCF10A was lower than that in MDA‐MB‐231 (ER‐) BC cells and MCF7 (ER+) BC cells (Figure 1C).

**FIGURE 1:**
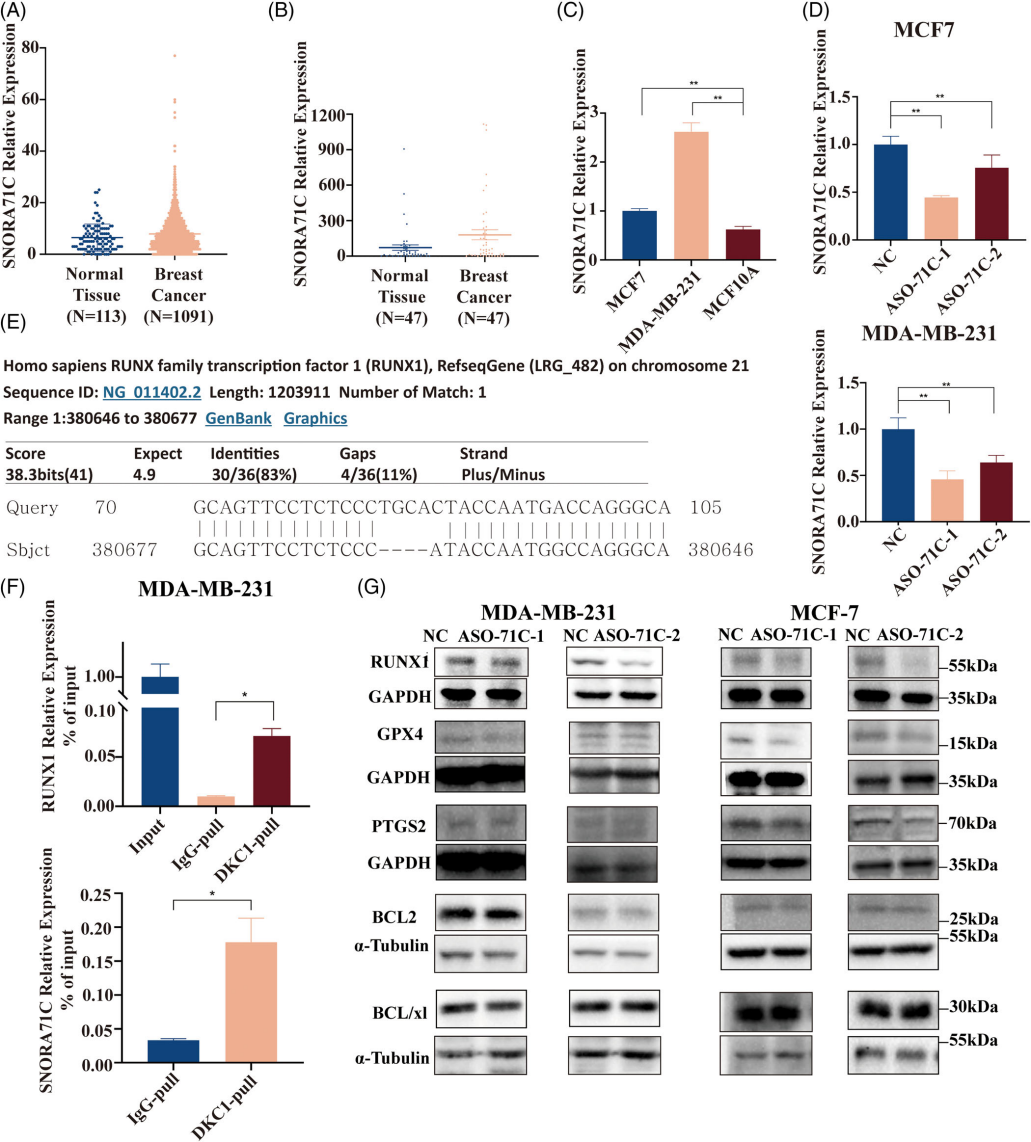
*SNORA71C* promotes development and metastasis of BC by regulating *RUNX1* and ferroptosis. (A) The difference expression of *SNORA71C* in BC and normal in The Cancer Genome Atlas (TCGA). (B) *SNORA71C* expression levels were detected using qRT‐PCR in BC tissues (*n* = 47) and normal tissue (*n* = 47). (C) Relative expression of *SNORA71C* in MCF10A, MCF7, and MDA‐MB‐231. (D) Level of *SNORA71C* in MDA‐MB‐231 and MCF7 cells, as determined by qRT‐PCR (normalized to U6) after treatment with ASO‐SNORA71C‐1 (antisense oligonucleotide SNORA71C‐1, ASO‐71C‐1) and ASO‐SNORA71C‐2 (antisense oligonucleotide SNORA71C‐2, ASO‐71C‐2). (E) The *RUNX1* variants of interest were confirmed using Sanger sequencing and BLAST. (F) RUNX1 was higher in the immunoprecipitate of DKC1 compared with control group. *SNORA71C* relative expression was higher in the immunoprecipitate of DKC1 compared with control group. (G) The expression of RUNX1, GPX4, and PTGS2 protein decreased after *SNORA71C* knocked down.

We designed and applied two antisense oligonucleotides (ASOs) targeting human *SNORA71C* to knock down the BC cell *SNORA71C* of MCF7 and MDA‐MB‐231, and confirmed by RT‐PCR that the expression level of *SNORA71C* was obviously knocked down after transfection of ASO‐SNORA71C in MDA‐MB‐231 and MCF7 cells (Figure 1D). Using Cell Counting Kit‐8 (CCK8), flow cytometry, wound healing and Transwell assay, we established that the proliferation ability of ASO‐SNORA71C transfected MDA‐MB‐231 and MCF7 cells, and the metastasis and invasion ability was significantly reduced, whereas the apoptosis rate was increased (Figure S1A–D).

Recent studies have shown that RNA‐binding protein can regulate the occurrence and development of cancer by binding to RNA and regulating its processing, stability, localization, modification, or translation. DKC1 is a structural protein of *SNORA71C*‐RBP complex and an important catalytic enzyme for pseudouracil modification of target mRNA. Runt‐related transcription factor‐1 (RUNX1) is involved in the occurrence and development of BC and plays an important role in the malignant behavior of tumors.^3^ Through gene sequence alignment, *SNORA71C* is found to match *RUNX1* by up to 83% (Figure 1E). RNA binding protein immunoprecipitation (RIP) results suggested that *RUNX1* mRNA was enriched in the complex of DKC1 and *SNORA71C* (Figure 1F). Knocking down *SNORA71C* can reduce the level of RUNX1 in MDA‐MB‐231 and MCF7 (Figure 1G). These results revealed that *SNORA71C* might promote the occurrence and development of BC through interaction with *RUNX1*. Earlier research has evidenced that RUNX1 exerts the opposite effect in BC^3^: it acts as a tumor inhibitor in ER‐positive (ER+) BC and is carcinogenic in ER‐negative (ER‐) BC (Figure S2A). In this study, Transwell and wound healing assay were performed to verify the conclusion above, after *RUNX1* knocked down in the ER+ cell line MCF7 and the ER‐ cell line MDA‐MB‐231 (Figure S2B,C). Our data showed that *SNORA71C* promotes tumor development in both MDA‐MB‐231 (ER‐) and MCF7 (ER+) BC cells (Figure S1A–D). Thus, we speculated that another more powerful carcinogenic mechanism might exist in ER+ MCF7 cells.

We established that after *SNORA71C* knock down, the proportion of cell death increased significantly, while no significant changes were observed in the levels of BCL family proteins, including BCL‐2 and BCL/XL in the classical apoptotic pathway (Figure 1G). Ferroptosis is a novel programmed cell death distinct from apoptosis, necrosis, and autophagy. It is characterized by iron‐dependent accumulation of lipid peroxides and is closely related to tumor occurrence, invasion, and metastasis. Prostaglandin‐endoperoxide synthase 2 (PTGS2) and glutathione peroxidase 4 (GPX4) are key proteins involved in the redox reaction during ferroptosis.^4^ Relevant literature has shown that GPX4 can promote tumor migration and invasion.^5^ We performed wound healing and Transwell assay after knocking down *GPX4* in MCF7 and MDA‐MB‐231 cell lines. It was found that the migration and invasion abilities of BC cells were significantly reduced (Figure S3A,B). Malondialdehyde (MDA) levels are typically used for the detection of membrane lipid peroxidation, and glutathione (GSH) is an important antioxidant and free radical scavenger in the body. Both are important indicators for the monitoring of the process of ferroptosis. Low levels of GSH have been associated with the ferroptosis occurrence. In the absence of GSH, the levels of free radicals and oxidation in the body increased, elevating MDA content. MDA and GSH assay could establish the occurrence of ferroptosis. After the *SNORA71C* knockdown in MCF7 (ER+) cells, the levels of GPX4 and PTGS2 decreased significantly (Figure 1G). MDA and GSH assay confirmed the occurrence of ferroptosis (Figure S4A,B).

However, the GPX4 and PTGS2 expression did not change in the MDA‐MB‐231 (ER−) cells (Figure 1G), nor did the MDA or GSH content (Figure S4A,B). These results indicated that *SNORA71C* only causes ferroptosis in ER+ BC cells. To verify whether PR can alter the effect of *SNORA71C*, we used PR+ MCF7 cells to knock down PR and detect the expression level of GPX4, PTGS2 protein, and the content of MDA, GSH, and GSSG in the cells (Figure S4C–E). The results showed no significant difference. Then, the expression level of GPX4 and PTGS2 protein and the content of MDA, total GSH, reduced GSH, and GSSG were detected by simultaneously comparing *SNORA71C* knockdown with PR and *SNORA71C* knockdown in PR+ MCF7 cells. We found no significant difference between the two groups, either (Figure S4C–E). Therefore, PR did not influence in the effect of *SNORA71C*. In summary, we speculate that *SNORA71C* promotes the proliferation of tumor cells associated with GPX4 and PTGS2, leading the reducing GSH increased and finally inhibiting ferroptosis in ER+ cells.

The founding of the present study showed that in BC we found that the role of *SNORA71C* was a complex, multidirectional, and involving a number of important biological processes. *SNORA71C* promoted the proliferation, migration, and invasion of ER‐ BC cells and inhibit apoptosis by combining with *RUNX1* in BC cells. Meanwhile, in ER+ BC cells, *SNORA71C* can increase the levels of PTGS2 and GPX4, elevated the content of GSH, suppressed the ferroptosis of ER+ BC cells, and performed a carcinogenic role. Therefore, *SNORA71C* could be used as a biomarker and a promising therapeutic target for BC treatment.

## AUTHOR CONTRIBUTIONS

Lin Zhao performed substantial contributions to conception and design, assembled the figure, and finally approved the version to be published. Bumin Xie performed the experiments, drafting the article or revising it critically for important intellectual content, provided technical and material support. At the time of revision, Xi Chen made great contributions during the process of data acquisition and analysis and subsequent manuscript content modification. All authors have read and approved the final manuscript.

### FUNDING INFORMATION

This study was supported by Natural Science Foundation of Liaoning Province (grant number: 20180551055).

### CONFLICT OF INTEREST STATEMENT

The authors declare no conflicts of interest.

## ETHICS STATEMENT

The Ethics Committee of Liaoning Cancer hospital and Institute approved the study (number: 20181228). Informed consent was obtained from all patients.

## Supporting information

Supporting Information

## Data Availability

The resources, tools, and codes used in our analyses were described in each method section in the methods. For any further of the data requests, please contact the corresponding author. The public datasets of TCGA analysed during the current study is: TCGA https://cancergenome.nih.gov/.
