# Supplementary material for: SNORA71C promotes development and metastasis of breast cancer by regulating RUNX1 and ferroptosis
Source: MedComm (2020). 2023 Apr 17;4(3):e262. doi: 10.1002/mco2.262 (PMC10106978; doi:10.1002/mco2.262)
Supplement: Supplementary file 1 — Supporting Information [file MCO2-4-e262-s001.docx]

**Supplementary Files**

**Title:** SNORA71C promotes the development and metastasis of breast cancer by regulating RUNX1 and ferroptosis

**Running Title:** SNORA71C acts as an oncogene in breast cancer

Author list: Bumin Xie^1#^, Xi Chen^1#^, Lin Zhao^1*^

^#^These authors contributed equally to this work.

^*^Correspondence to: Lin Zhao, szl_zhaolin@126.com

**Authors’ affiliations:**

1 Department of Breast Surgery, Cancer Hospital of Dalian University of Technology, Liaoning Cancer Hospital & institute, Shenyang, Liaoning Province, 110042, People's Republic of China;

**Supplementary Methods and** **Materials**

**Data analyses and bioinformatics**

To analyze the differential expression of snoRNAs in breast cancer tissue using The Cancer Genome Atlas (TCGA) database (<https://cancergenome.nih.gov/>), a T test (P < 0.05) combined with fold change (FC) was used to identify the differentially expressed snoRNAs. (log2 (FC) ≥ 2 for upregulated snoRNAs)

**Cell culture and treatment**

MDA-MB-231, MCF7 breast cancer cells and human breast epithelial cell MCF10A were purchased from the ATCC (American Type Culture Collection, Manassas, VA, USA). MDA-MB-231 and MCF7 cells were cultured in Roswell Park Memorial Institute (RPMI) 1640 medium or Dulbecco’s modified Eagle’s medium (DMEM) with 10% fetal bovine serum, 100 U/ml penicillin, and 100 μ g/ml streptomycin. Cells were incubated with 5% CO_2_ at 37 °C. MCF10A were cultured in MEGM (Lonza) supplemented with 5% horse serum, 0.5μg/mL hydrocortisone, 20ng/mL EGF, 5μg/mL insulin, 100 ng/mL cholera toxin, and antibiotic. According to the recommended culture conditions, the medium was changed every two or three days. When the cell confluence reached 80%, the cells were digested with trypsin (0.1% trypsin). According to the manufacturer's protocol, Lipofectamine 3000 reagent (Invitrogen, Carlsbad, CA, USA) was used to enable cell transfection. Antisense oligonucleotides (ASOs) were transfected into breast cancer cells. The antisense oligonucleotide sequence was ASO-h-SNORA71C_ 001: GGAACTGCCGAGAGCACTTC. ASO-h-SNORA71C_ 002: AATGACCAGGGCACGGGCA

**Cell Counting Kit-8 (CCK-8) assay**

The proliferation of MDA-MB-231 and MCF7 breast cancer cells transfected with ASO-SNORA71C was determined using a CCK-8 assay (Yeasen, Shanghai, China). 2000 cells in 100 μl of medium were inoculated into each well of a 96-well plate and incubated until adhesion. Then, 100 nM ASO‑SNORA71C or control ASO-NC (negative control ASO) were transfected into the cells. At 0, 24, 48, 72, and 96 h time points, 10 μl of CCK-8 solution was added to each well and incubated for 3 h. The absorbance at 450 nm was then measured using a microplate spectrophotometer (BioTek Instruments, Winooski, VT, USA).

**Invasion assay**

Matrigel (Becton Dickinson Labware, Bedford, MA, USA) was diluted 1 : 15 in serum-free medium to a final concentration of 8 mg/ml, and 40 μl was spread evenly on the membrane lining the upper chamber of Transwell inserts (BD Bioscience, San Jose, CA, USA). It took 4 h to the Matrigel to set at 37°C. Then cells were seeded onto the filters at a concentration of 5× 10^4^ cells/well in 200 μL of FBS‐free medium and then transfected. The lower chambers were filled with 600 μL of medium with 10% FBS. After 48 hours of incubation, the Transwell inserts were removed, gently washed with PBS twice, fixed with 4% paraformaldehyde for 30 minutes, washed twice with PBS again, and stained with 0.1% crystal violet. Then the Matrigel layer was wiped off to remove the noninvasive cells. The membranes were mounted on a glass slide after cut out from the inserts.

**Flow cytometry assay**

Flow cytometry (BD Biosciences, San Jose, CA, USA) was used to detect the apoptosis of cells stained with PI and annexin v-fluorescein isothiocyanate (FITC) (BD Biosciences, San Jose, CA, USA) according to the manufacturer’s instructions. Briefly, after incubation in 5% CO2 at 37℃ for 72 hours, the cells were washed three times with cold PBS and stained with PI and FITC solution. The samples were gently vortex and incubated before flow cytometry was performed.

**Wound-healing assay**

1 × 10^6^ (monolayer) cells were seeded in 6-well culture plate for incubation. After the cells adhered to the wall, the fused monolayer was scratched using a pipette tip, washed, and cultured in medium containing 10% FBS. The scratch was photographed at 0, 24, 48, and 72 h; Image J software (National Institutes of health, Bethesda, MD, USA) was used to measure the wound area. The wound healing rate = (original wound area − actual wound area at different times)/original wound area × 100%。

**Normal breast tissue and breast cancer specimens**

From August 2003 to December 2011, normal breast tissues and breast cancer tissues were obtained from patients at the Liaoning Cancer hospital and Institute (Liaoning, Shenyang, China). Samples were obtained, immediately frozen in liquid nitrogen, and stored frozen until analysis. No chemotherapy, radiotherapy, or adjuvant therapy was administered before surgery. All subjects provided informed consent. The medical ethics committee of Liaoning Cancer hospital and Institute approved the study. (No.20181228)

**Quantitative real-time reverse transcription PCR (qRT-PCR)**

Total RNA was extracted from breast cancer cell lines, normal breast tissue, and breast cancer tissues using TRIzol (Takara, Shiga, Japan). Total RNA (2 μg) was reverse transcribed to cDNA and the cDNA was the used as a template for qPCR reactions. The relative expression of mRNA was compared with that of reference gene *GAPDH* (encoding glyceraldehyde-3-phosphate dehydrogenase) or U6. The oligonucleotide primers used in qPCR were based on sequences obtained from GenBank. The relative RNA expression was calculated using the 2- ΔΔCt method.

**Western blotting**

Cells were lysed on ice for 30 minutes and then centrifuged at 4 °C for 15 minutes. The supernatant was retained and a Bicinchonic acid (BCA) quantification Kit (Beyotime Biotech Inc., Shanghai, China) was used to measure the total protein of each group. The protein samples were separated by sodium dodecyl sulfate polyacrylamide gel electrophoresis (SDS-PAGE). The separated proteins were transferred to a polyvinylidene difluoride (PVDF) membrane, incubated with 3% bovine serum albumin at room temperature, and then incubated with primary antibodies at 4 °C overnight. The membrane was washed with Tris-buffered saline-Tween 20 (TBST) five times for 10 min each time. After incubation with secondary antibody (1:8000) (Proteintech, SA00001-2) at room temperature for 2 h, the membrane was washed three times with TBST for 5 min each time. Then, electrochemiluminescence imaging was performed using the ECL system.

RUNX family transcription factor 1 (RUNX1) (Proteintech, Rosemont, IL, USA; 25315-1-ap), prostaglandin-endoperoxide synthase 2 (PTGS2) (Proteintech, 66351-1-ig), glutathione peroxidase 4 (GPX4) (Proteintech, cl488-67763), B-cell CLL/lymphoma 2 (Bcl2) (Proteintech, 12789-1-AP), Apoptosis regulator Bcl X(Bcl-XL) (Proteintech, 26967-1-AP), Glyceraldehyde-3-phosphate dehydrogenase (GAPDH) (Proteintech, 60004-1-Ig), Glyceraldehyde-3-phosphate dehydrogenase (GAPDH) (Proteintech, 10494-1-AP), Alpha Tubulin (Proteintech, 11224-1-AP), Alpha Tubulin (Proteintech, 66031-1-Ig).

**RNA binding protein immunoprecipitation (RIP)**

Cells were collected and lysed with RIP lysis buffer (Beyotime Biotech Inc.) containing ribonuclease (1000 U/ml) and deoxyribonuclease I (50 U/ml). After centrifugation at 12000 g for 15 minutes, the supernatant was removed and cultured overnight with anti-dyskerin pseudouridine synthase 1 (DKC1) antibodies (Abcam, Cambridge, MA, USA; ab93777) or IgG (Proteintech, b900610) at 4 °C. Protein A/G beads (B23202, Bimake, Huissen, Netherlands) were added and cultured at room temperature for 4 h. After washing the beads with elution buffer, the immunoprecipitated RNA was purified using Trizol, ethanol precipitated, and analyzed using qRT-PCR.

**Malondialdehyde (MDA) assay**

The cells were collected and lysed with RIP lysis buffer. The supernatant was collected after centrifugation at 12000 g for 15 minutes. A small amount of supernatant was absorbed, and the total protein of each group was measured using the BCA Kit. The experimental group, the control group, and the blank sample were added with 0.1 ml MDA detection working solution, respectively. Separately, a calibration curve was constructed using MDA concentration standards of 1, 2, 5, 10, 20, 50 μM. After mixing, the samples were placed in water bath at 100 °C for 15 minutes. After colling to room temperature, the samples were centrifuged at 1000 × *g* at room temperature for 10 minutes. Then, 0.2 ml of the supernatant was added to wells of a 96-well plate and the absorbance at 532 nm was measured using a microplate reader. The MDA content was calculated with the protein content.

**Glutathione (GSH) and oxidized glutathione (GSSG) assays**

Cells were collected and divided into 2 groups equally. According to the manufacture`s instruction, the cells of the first group were lysed and used for BCA detection to measure the total protein in each group. The cells of the other group were added into three volumes (relative to the pelleted cells) of protein removal reagent M solution and mixed well. The samples were frozen and thawed twice using liquid nitrogen and a 37 °C water bath. Finally, the samples were placed in an ice bath for 5 min and centrifuged at 4 °C for 10 min at 10000 × *g*. Half of the supernatant was used to determine the content of total GSH. 20 μl of diluted GSH scavenging auxiliary solution was added to the samples and mixed immediately. Then, 4 μl of GSH scavenging working solution was added and mixed immediately. The reaction was carried out at 25 °C for 60 minutes. Then, 10 μl of the GSH and GSSG samples were added separately to 150 μl of total glutathione detection working solution and reacted at 25 °C for 5 minutes. Then, 50 μL 0.5 mg/ml NADPH and mixed well. After reacting for 25 min, the absorbance was measured at 405nm and the GSH and GSSG levels were calculated.

**Statistical analysis**

All data were analyzed using GraphPad Prism 6.0 (GraphPad Inc., La Jolla, CA, USA). A two tailed t‑test was used to analyze the differences between two groups. Differences with p < 0.05 were considered statistically significant.

**
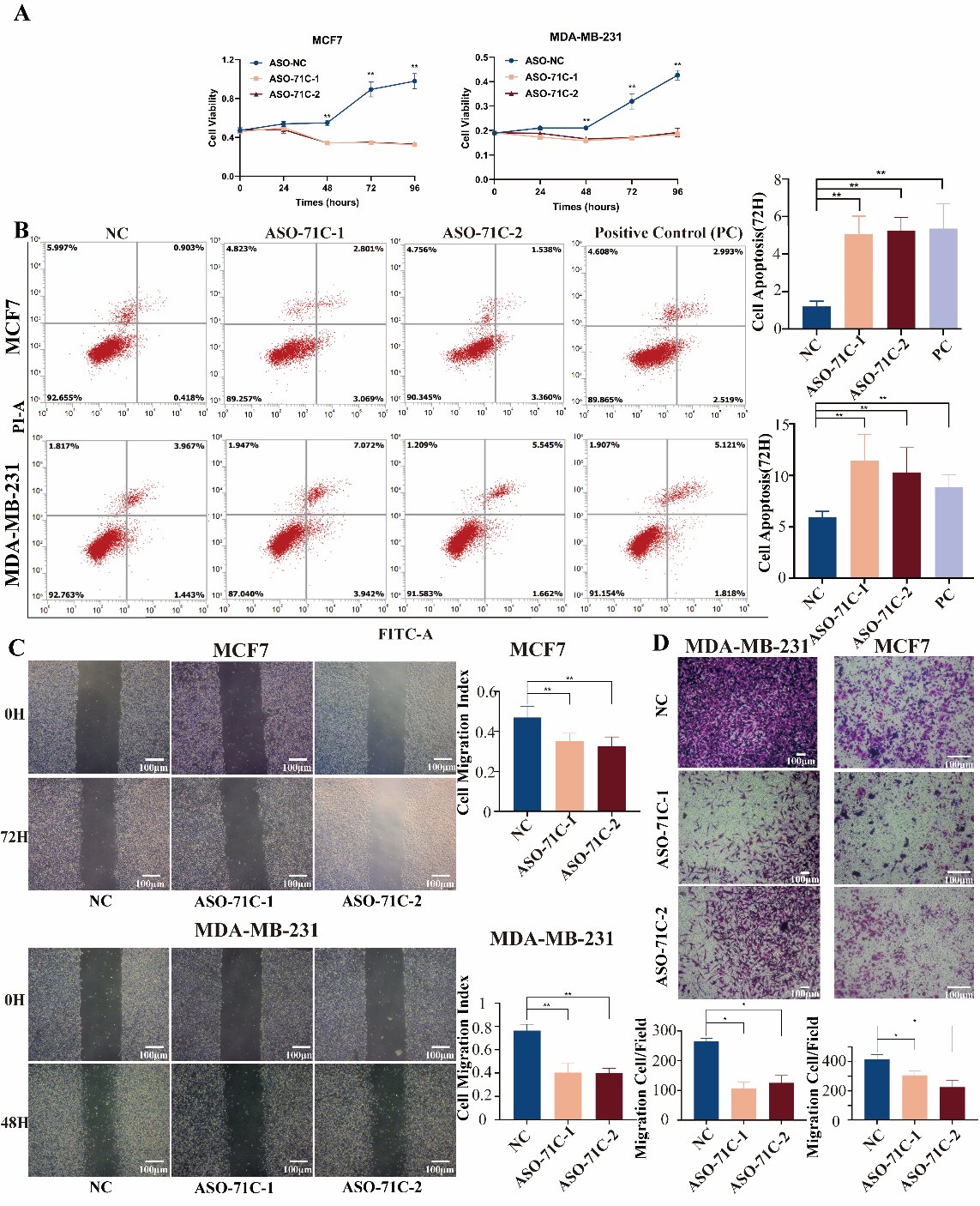
**

**Figure Supplement 1**

The cell proliferation, migration, and invasion were promoted and the apoptosis rate was reduced after *SNORA71C* knocked down in MDA-MB-231 and MCF7. **A** CCK8 assay was used to detect cell proliferation capability. **B** Detection of the apoptosis of MDA-MB-231 and MCF7 cells by flow cytometry assay. Erastin is used as positive control (PC). **C** Migration capability of MDA-MB-231 and MCF cells was detected by cell scratch assay. **D** Invasion capability of MDA-MB-231 and MCF cells was detected by cell Transwell assay. Data is shown as the mean ± SD (error bars) from more than three independent repeats. *P < 0.05, **P < 0.01 (Student’s t-test).


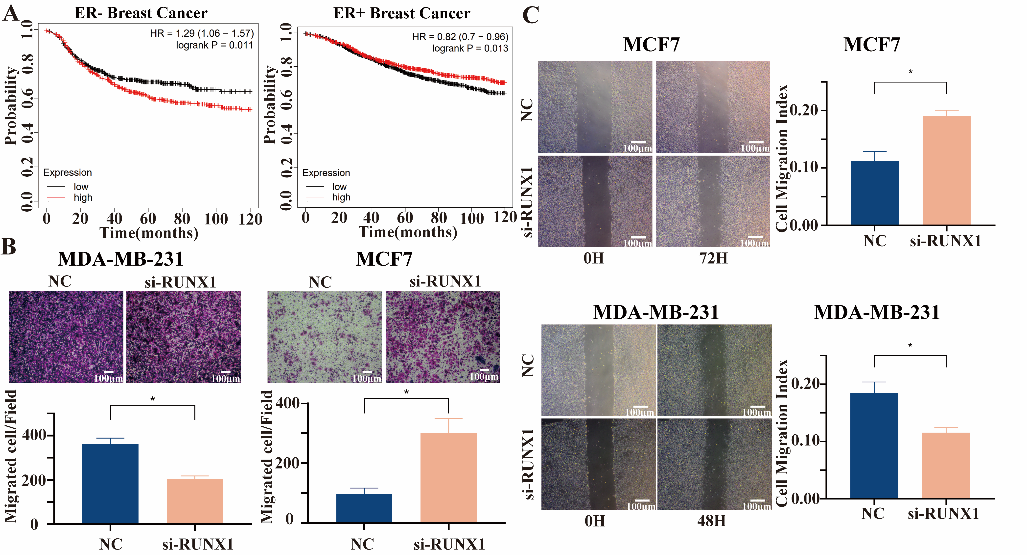


**Figure Supplement 2**

*RUNX1* is related to poor prognosis in ER- breast cancer tissues, while in ER+ tissues, *RUNX1* acts as a protective prognostic indicator. **A** *RUNX1* acts as a tumor inhibitor in estrogen receptor-positive (ER+) BC and is carcinogenic in estrogen receptor-negative (ER-). **B** Invasion capability of MDA-MB-231 and MCF cells was detected by cell Transwell assay after *RUNX1* knockdown. **C** Migration capability of MDA-MB-231 and MCF cells was detected by cell scratch assay after *RUNX1* knockdown.


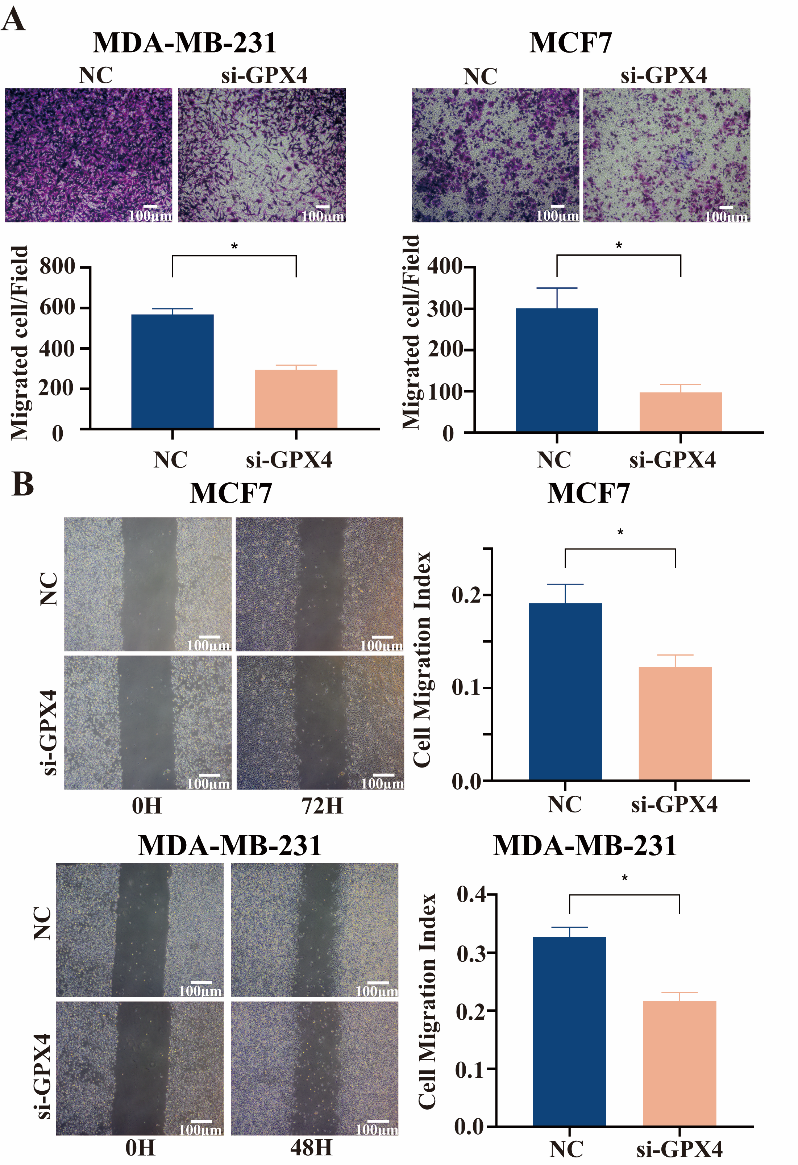


**Figure Supplement 3**

SiRNA targeting GPX4 inhibits cell migration and invasion in MDA-MB-231 and MCF7. **A** Invasion capability of MDA-MB-231 and MCF cells was detected by cell Transwell assay after RUNX1 knockdown and GPX4 knockdown. **B** Migration capability of MDA-MB-231 and MCF cells was detected by cell scratch assay after GPX4 knockdown.

**
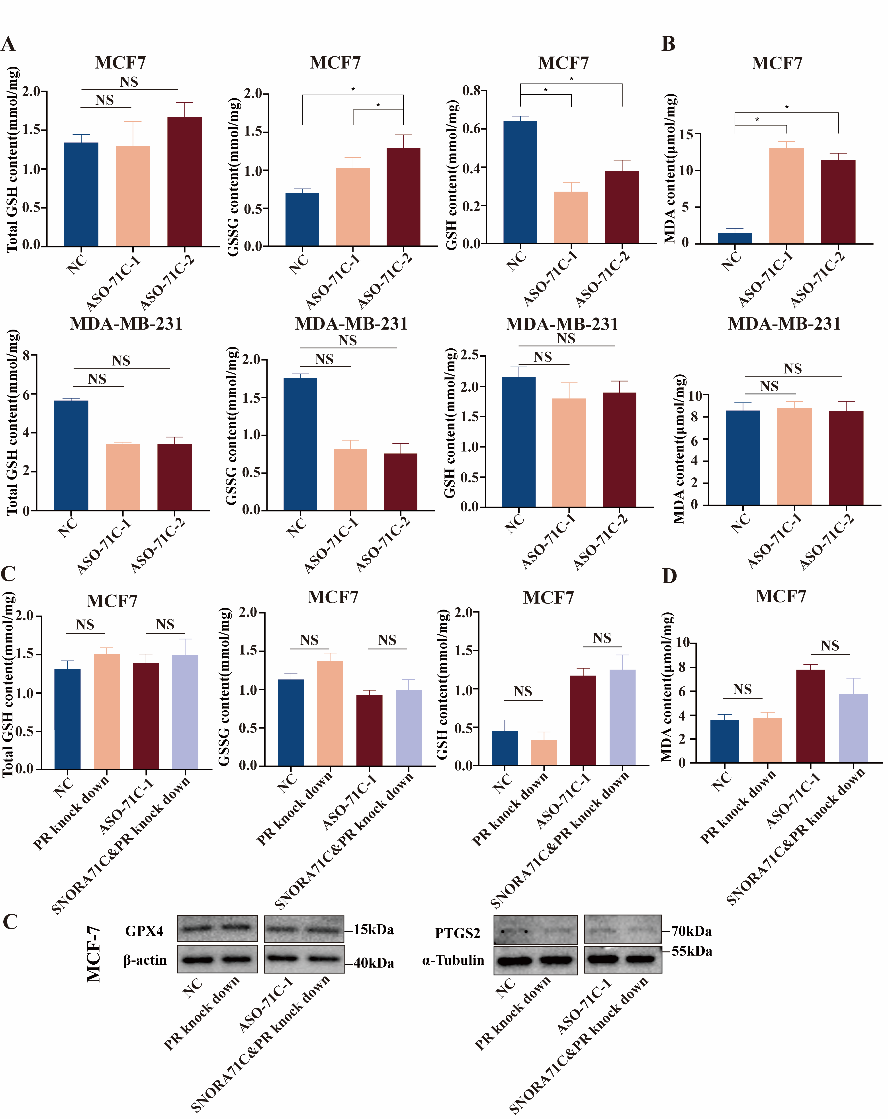
**

**Figure Supplement 4**

The GSH and MDA content decrease after *SNORA71C* knock down in MCF7 and MDA-MB-231. PR would not influence the effect of SNORA71C. **A** Detection of GSH and GSSG level of MCF7 cells and MDA-MB-231 cells. **B** MDA level of MCF7 cells and MDA-MB-231. **C** MDA level of MCF7 cells after *SNORA71C* knockdown, PR knockdown, and PR knockdown with *SNORA71C* knockdown simultaneously. **D** Detection of GSH and GSSG level of MCF7 cells after *SNORA71C* knockdown, PR knockdown, and PR with *SNORA71C* knockdown simultaneously. **E** The expression of GPX4 and PTGS2 was detected after *SNORA71C* knockdown, PR knockdown, and PR with *SNORA71C* knockdown simultaneously in MCF7 cells. NS represents no significant.

**Supplementary Table**

**Table S1:** Correlation of SNORA71C expression with different clinicopathological features of breast cancer

| Patients’ frequency (%) | | SNORA71C expression | | *P*-value |
| --- | --- | --- | --- | --- |
|  |  | High expression | Low expression |  |
|  | Total N=47 |  |  |  |
| Age(years) |  |  |  |  |
| ≤50 | 13 | 6 | 7 | 0.4423 |
| >50 | 34 | 17 | 17 |  |
| Tumor size(cm) |  |  |  |  |
| ≤3.0 | 40 | 22 | 18 | 0.3832 |
| >3.0 | 7 | 1 | 6 |  |
| LN metastasis |  |  |  |  |
| Negative | 21 | 12 | 9 | 0.3830 |
| Positive | 26 | 11 | 15 |  |
| Ki67 |  |  |  |  |
| ≤30% | 18 | 10 | 8 | 0.3955 |
| >30% | 29 | 13 | 16 |  |
| ER |  |  |  |  |
| Negative | 16 | 9 | 7 | 0.3285 |
| Positive | 31 | 14 | 17 |  |
| PR |  |  |  |  |
| Negative | 25 | 14 | 11 | 0.0921 |
| Positive | 22 | 9 | 13 |  |
| HER2 |  |  |  |  |
| Negative | 18 | 6 | 12 | 0.1749 |
| Positive | 29 | 17 | 12 |  |
| Class |  |  |  |  |
| Class 1 | 5 | 2 | 3 | Class 1 vs Class 2 0.3459 |
| Class 2 | 35 | 17 | 18 | Class 2 vs Class 3 0.4785 |
| Class 3 | 7 | 4 | 3 | Class 1 vs Class 3 0.3323 |
| TNM Stage |  |  |  |  |
| I + II | 42 | 20 | 22 |  |
| III + IV | 5 | 3 | 2 | 0.4330 |

**Table S2**. Primer sequences in Q-PCR

| Genes | Forward Primer | Reverse Primer |
| --- | --- | --- |
| *SNORA71C* | CGTGCCCTGGTCATTG | GAGTGGACCCTCCAAACA |
| *RUNX1* | TCAGGTTTGTCGGTCGAAG | GCCCATCCACTGTGATTTTG |
| *GPX4* | GTGGATGAAGATCCAACCC | TTGTCGATGAGGAACTTGG |
| *PR* | CGTACCCTCTCTATAGCGACTT | ACCGGCCACAAGGTAGGAA |

^*^ All the Primers are provided by BGI Genomics
